# Development and Validation of the Common Prosperity Aspiration Scale: A Mixed-Methods Study in China

**DOI:** 10.3390/bs16020203

**Published:** 2026-01-30

**Authors:** Huicun Duan, Qinglong Guo, Jingfeng Han, Na Chen, Hong Chen

**Affiliations:** 1 Faculty of Psychology, Southwest University, Chongqing 400715, China; dhc688795@email.swu.edu.cn (H.D.); expound@email.swu.edu.cn (Q.G.); chenna12@swu.edu.cn (N.C.); 2 Research Center of Psychology and Social Development, Southwest University, Chongqing 400715, China; 3 School of Psychology, Shandong Normal University, Jinan 250358, China; hanjinfeng@sdnu.edu.cn; 4 Key Laboratory of Cognition and Personality (SWU), Ministry of Education, Chongqing 400715, China; 5 Center for Studies of Education and Psychology of Ethnic Minorities in Southwest China of Southwest University, Chongqing 400715, China; 6 Collaborative Innovation Team for Children and Adolescent Mental Health, Southwest University, Chongqing 400715, China

**Keywords:** common prosperity aspiration, rural development, community psychology, mixed-methods research, scale development

## Abstract

Despite the increasing emphasis on residents’ prosperity aspirations in rural development initiatives, the lack of a psychometrically sound measure limits comparability and rigor, as existing studies primarily focus on structural and policy factors influencing community prosperity, with insufficient attention to residents’ psychological processes and subjective experiences. Drawing on community psychology, this study develops and validates a measure of rural residents’ aspirations for common prosperity, integrating personal fulfillment with collective advancement across material and spiritual domains. Employing a three-phase mixed-methods design, Study 1 used in-depth interviews and grounded theory procedures (*N* = 28) to develop a theoretical model comprising four dimensions: material–individual, material–collective, spiritual–individual, and spiritual–collective. Study 2 generated a 19-item, four-factor scale via exploratory factor analysis and exploratory graph analysis (*N* = 581). Study 3 confirmed the scale’s second-order factor structure and psychometric properties with confirmatory factor analysis (*N* = 659). The Common Prosperity Aspiration Scale (CPAS) demonstrated strong reliability and validity across its four dimensions and the overarching second-order factor. This pioneering study elucidates the psychological structure of common prosperity aspirations and provides a psychometrically reliable measure for rural contexts. It serves as a valuable tool to explore their influence on behaviors and promote sustainable community development.

## 1. Introduction

Rural China has undergone profound political, economic, and social transformations since the founding of the People’s Republic in 1949, shaping residents’ aspirations in line with cultural ideals of collective well-being. Politically, it evolved from collectivization in the 1950s–1970s, which fostered communal harmony through centralized agricultural production, to the 1978 Household Responsibility System, empowering households while preserving collective governance, and now features village-level self-governance (Frazier, 2010; Gurley, 1975). Economically, areas transitioned from pre-reform poverty to comprehensive alleviation by 2021, lifting nearly 800 million people out of extreme poverty through targeted policies emphasizing modernization, digital villages, and sustainable development, rooted in traditional values like diligence and harmony, while addressing ongoing dynamics like land management and labor mobility (Dunford, 2022; National Bureau of Statistics of China, 2025; World Bank & Development Research Center of the State Council, the People’s Republic of China, 2022). Regarding migration, the hukou (household registration) system has guided mobility by maintaining rural ties and family networks, drawing on Confucian filial piety; recent reforms under new-type urbanization have facilitated integration, enabling remittances for collective village goals (Chan & Zhang, 1999; Wing Chan & Buckingham, 2008). These transformations, intertwined with cultural traditions, have advanced material and collective prosperity (Lan & Jin, 2025a; Seeberg, 2024; Waight et al., 2025).

China’s Common Prosperity (*Gongtong Fuyu*, 共同富裕) strategy outlines a transformative vision for achieving equitable wealth distribution, cultural enrichment, and spiritual well-being by 2035 (A. Hu et al., 2025). Rooted in socialist principles, this strategy embodies the ideological foundation of Chinese socialism and the collective aspirations of its people (Y. Wang et al., 2022). Central to this agenda is rural revitalization, which serves as a critical pathway to advance the economic, cultural, and social development of approximately 465 million rural residents (National Bureau of Statistics of China, 2025). By fostering comprehensive rural development, Common Prosperity aims to bridge urban-rural disparities and promote broader societal harmony.

Despite growing emphasis on the Common Prosperity strategy, rigorous psychological studies exploring CPA among rural residents remain limited. Existing research primarily focuses on structural factors, such as infrastructure and economic policies (Yang et al., 2023), with limited attention to the subjective experiences and psychological drivers of rural residents. Some studies have examined residents’ perceptions of community development, revealing mixed outcomes: for instance, grounded theory approaches show that participatory governance enhances community happiness but can exacerbate inequality and management confusion (D. Wang et al., 2025), while tourism-led development fosters supportive attitudes when benefits are equitably shared, yet unequal distribution erodes collective motivation (Dang & Li, 2025). These works extend empowerment and Sustainable Livelihoods frameworks to Chinese contexts but largely overlook underlying individual and collective motivational aspirations, especially in collectivist settings where shared prosperity could bolster community efficacy.

Overall, few studies have systematically investigated the interplay between individual and collective dimensions of CPA or its behavioral and policy implications, creating a significant gap in which psychological factors are crucial for community engagement and policy effectiveness. To address this critical gap, this study employs a community-centered, mixed-methods approach to develop and validate the Common Prosperity Aspiration Scale (CPAS), offering a theoretically and empirically grounded tool to explore CPA in rural contexts and support sustainable community development.

### 1.1. Aspiration

Aspiration and desire, while related psychological constructs, differ in their temporal orientation and social scope. Desires typically represent immediate wants or needs driven by biological or psychological drives. In contrast, aspirations refer to future-oriented goals that transcend immediate circumstances and often encompass broader social meanings (Locke & Latham, 2002). This distinction makes aspiration particularly relevant for understanding Common Prosperity, which inherently involves long-term, socially embedded goals that extend beyond individual gratification.

Psychological research provides a foundation for understanding aspiration through multiple theoretical lenses. Freud conceptualized desires as fundamental motives driving unconscious behavior (Freud, 2016), while subsequent research defined aspirations as idealized goals unconstrained by immediate reality (King, 1995). Wellman and Bartsch (1989) proposed a belief-desire reasoning model from cognitive psychology, suggesting that behaviors are interpreted through desires and beliefs, extending to aspirations as conscious pursuits of future-oriented goals. Contemporary research categorizes aspirations into intrinsic, achievement, and instrumental types, with intrinsic aspirations linked to autonomy and relatedness fostering collective goal pursuit (Schroeder, 2004; Hart, 2016).

From a community psychological perspective, socioeconomic factors significantly influence individuals’ capacity to form and pursue prosperity-related aspirations. Research demonstrates that higher socioeconomic status correlates with psychological well-being and life satisfaction, as resource access enables engagement in novel activities (Oishi et al., 2020). Conversely, lower SES individuals may experience constrained aspiration formation due to resource limitations and reduced perceived opportunities (Guo et al., 2015; Kraus et al., 2017). Additionally, prosocial behavior studies show that altruistic acts activate reward centers (Harbaugh et al., 2007), suggesting that collective well-being aspirations may provide intrinsic psychological rewards, particularly relevant for understanding CPA.

### 1.2. Common Prosperity in Interdisciplinary Contexts

Existing research predominantly examines aspirations within Western individualistic frameworks, potentially limiting applicability to China’s collective contexts. Common Prosperity encompasses both material and spiritual dimensions (Xi, 2021), operating within a collectivist cultural framework where individual and collective well-being are intertwined. This necessitates examining how aspirations function when targeting shared prosperity goals rather than purely individual achievements, particularly in rural Chinese contexts where community interdependence remains strong.

Interdisciplinary research on common prosperity highlights its multifaceted nature but reveals a psychological gap. Economic studies focus on regional disparities and labor market dynamics, advocating policy adjustments to reduce urban-rural gaps (Dunford, 2022). Political science research emphasizes governance structures and institutional reforms to support common prosperity (Zheng, 2024). Sociological studies explore social mobility and perceived pressures, revealing how structural factors shape residents’ expectations (Lan & Jin, 2025b). Y. Wang et al. (2022) developed a social mentality index for common prosperity, noting high public confidence but low participation, suggesting a disconnect between policy goals and community engagement. These studies provide critical structural and social insights, often overlooking micro-psychological processes shaping CPA.

Psychological research on well-being, such as Diener’s subjective well-being and flourishing scales (Diener, 1984; Diener et al., 2010), offers individual welfare assessment tools but lacks collective focus relevant to common prosperity’s shared development emphasis. E. Zhang et al. (2023) developed a comprehensive evaluation model integrating prospect theory with probabilistic linguistic approaches, successfully applied to Zhejiang Province, China’s designated common prosperity demonstration zone. However, this framework focuses primarily on systematic measurement rather than underlying psychological dimensions of collective well-being and spiritual fulfillment, highlighting the need for a construct integrating individual and collective, material and spiritual aspirations within China’s collectivist cultural context.

### 1.3. Common Prosperity Aspiration

CPA is conceptualized as context-specific psychological motivations of rural residents for achieving shared material and spiritual well-being in community settings. This definition addresses the need for a psychological construct integrating personal aspirations with communal goals in collectivist society, grounded in China’s national Common Prosperity strategy emphasizing equitable development (A. Hu et al., 2025; Xi, 2021).

Drawing on Bronfenbrenner’s ecological systems theory (Bronfenbrenner, 1981, 1994) and community psychology principles (McMillan & Chavis, 1986), CPA reflects the dynamic interplay between individual factors (e.g., personal motivations) and environmental influences at the micro-level (e.g., family and community interactions) and macro-level (e.g., cultural norms and national policies). In rural China, collectivist values and traditional social structures, such as Fei’s “differential mode of association (relationship networks based on kinship and social proximity)” (Fei, 1992), shape aspirations for both individual achievement and collective well-being. This multidimensional construct encompasses material (e.g., economic security) and spiritual dimensions (e.g., cultural fulfillment), as well as individual and collective orientations. This distinguishes CPA from Western individual-centric constructs like subjective well-being that focus on current emotional states rather than future-oriented motivations (Diener, 1984). Rather than representing happiness or well-being itself, CPA represents the motivational drive toward shared prosperity, bridging personal desires with community-oriented outcomes in collectivist contexts.

The absence of standardized measurement tools limits systematic exploration of CPA and its application in policy design. Existing scales focus on individual well-being and fail to capture the collective and culturally specific nature of common prosperity. A psychometrically robust scale would enable assessment of individual differences, facilitate cross-community comparisons, and inform resident-centered interventions.

### 1.4. The Current Study

This research aims to develop the CPAS, a psychometrically robust, multidimensional self-report instrument measuring rural residents’ CPA. The theoretical framework integrates community psychology principles emphasizing collective efficacy and community belonging with ecological systems theory, analyzing multi-level environmental influences on aspirations. To ensure rigorous investigation, we employ a multi-stage mixed-methods design incorporating qualitative interviews and quantitative exploratory and confirmatory analyses across three studies, ensuring comprehensive examination of CPA’s conceptual foundation, dimensional structure, and measurement validity informed by residents’ experiences and aspirations.

## 2. Study 1: Exploring the Sources and Structure of Common Prosperity Aspiration

Study 1 employed grounded theory to explore the psychological constructs of common prosperity aspiration among rural Chinese residents. Participants were recruited from both village cadres (interviewed in dual roles as officials and residents) and ordinary villagers, to capture regional and role-based variations. Analysis of semi-structured interview data using NVivo 14 revealed a two-dimensional four-quadrant theoretical model that integrates material versus spiritual and individual versus collective orientations, providing a culturally grounded operationalization of common prosperity aspiration in rural contexts.

### 2.1. Methods

#### 2.1.1. Participants

This study received ethical approval from the Institutional Review Board (IRB; Approval No. H21092), and informed consent was obtained from all participants. To protect participant confidentiality in accordance with IRB guidelines, specific locations are described only using broad regional descriptors. A total of 28 rural residents were recruited from two contrasting rural regions: one in an eastern coastal province and the other in a western inland province. These regions were selected to include both areas actively implementing common prosperity initiatives (such as the national demonstration zone) and comparable non-demonstration areas. The sample included both Han and ethnic minority participants and was designed to ensure diversity across community roles, genders, ages, and ethnicities. Participants were recruited in two phases:

Phase 1: 15 participants from a western inland region (non-demonstration area; 8 village cadres, 7 residents), aged 25–57 years (8 males, 7 females; 6 minority participants).

Phase 2: 13 participants from an eastern coastal region (demonstration area; 3 village cadres, 10 residents), aged 27–63 years (7 males, 6 females; 2 minority participants).

Demographic characteristics, subjective economic status (assessed via the MacArthur Scale of Subjective Social Status; Adler et al., 2000), and annual per capita disposable income are summarized in Table 1.

#### 2.1.2. Data Collection and Interview Process

Theoretical sampling guided participant recruitment across two phases until theoretical saturation was achieved (see Section 2.1.3 for saturation details). Adopting a grounded theory approach (Corbin & Strauss, 2008), this study purposively recruited participants from two contrasting rural regions: one in an eastern coastal province and the other in a western inland province (see Section 2.1.1). For village cadres, a dual-identity approach was employed: they were first interviewed in their official governance role and then as ordinary residents, minimizing role-related bias and enriching perspectives on governance and daily life. Data collection proceeded in two phases: Phase 1 (October–November 2023) involved face-to-face interviews, whereas Phase 2 (December 2023–February 2024) used online interviews via *Tencent Meeting*. The process included a preliminary stage followed by a formal stage. In the preliminary stage, semi-structured interviews were conducted with 5 rural residents (not included in the final sample) to gain initial insights into their perceptions of common prosperity. These interviews informed the refinement of a formal interview guide. In the formal stage, semi-structured interviews were conducted with the 28 participants described in Section 2.1.1. Interviews lasted 35–68 min, and generated approximately 250,000 words of transcripts. The interview guide addressed four main areas: (1) perceptions and expectations of common prosperity, (2) motivations for pursuing common prosperity, (3) experiences and reflections during the pursuit, and (4) visions for future community development. All interviews were transcribed within 24 h, verified for accuracy by a second researcher, and subjected to member checking with selected participants to confirm interpretations.

#### 2.1.3. Data Analysis

Grounded theory was applied to analyze the interviews, allowing constructs to emerge directly from data (Corbin & Strauss, 2008). This approach was selected because the common prosperity aspiration lacked a clear operational definition at the time. The analysis followed three iterative steps:

Open Coding: Using NVivo 14 software, key meaning units were identified, summarized into initial nodes, and categorized with detailed conceptual descriptions. This initial phase focused on breaking down raw data into discrete concepts, ensuring cultural sensitivity to rural Chinese community contexts by incorporating community psychology principles.

Axial and Selective Coding: Nodes were refined through homogeneous and heterogeneous comparisons, linking main and sub-categories to construct higher-level concepts and clarify hierarchical structures. Regional variations were identified and integrated via constant comparative analysis across regions (Corbin & Strauss, 2008), balancing contextual differences with the core commonalities of the theoretical model. To ensure transparency and prevent arbitrary classification, hierarchical distinctions were guided by explicit coding rules derived inductively from the data and anchored in established theories: Maslow’s (1954) hierarchy of needs for the material-to-spiritual progression (lower-order needs, e.g., survival and security, versus higher-order aspirations, e.g., belongingness and self-actualization), and Markus and Kitayama’s (1991) interdependent self-construal theory for the individual-to-collective orientation (independent self-construal emphasizing personal autonomy and individual fulfillment versus interdependent self-construal focused on relational harmony and collective prosperity). These rules formed a transparent coding rubric detailed in Table S1 (Supplementary Materials), including specific decision criteria for assigning meaning units to levels and dimensions. Logical relationships between nodes were analyzed to derive core dimensions of common prosperity aspiration, and a theoretical model was developed through iterative validation against the original data.

Finally, theoretical saturation was assessed following established grounded theory guidelines (Corbin & Strauss, 2008). We considered saturation achieved when no new codes or themes emerged, existing categories were richly illustrated with diverse examples, and relationships among categories were consistent across additional interviews. By the 21st interview, these criteria were met. To confirm robustness, seven additional interviews were conducted (three in the western inland region and four in the eastern coastal region), all of which demonstrated complete redundancy with established codes and categories, providing no new insights relevant to the research questions.

### 2.2. Reliability and Validity

#### 2.2.1. Reliability

To ensure reliability, a comprehensive approach incorporating multiple validation strategies was employed (Flick, 2007). All interviewers completed standardized training in semi-structured interview methodology and qualitative data analysis protocols using NVivo software. The pilot phase involved conducting preliminary interviews with 5 villagers (not included in the final sample) to gather initial insights into their perceptions and aspirations for common prosperity. Feedback from these pilot interviews, including participants’ comments on question clarity and relevance as well as interviewers’ reflections on interview flow and probing techniques, informed refinement of the interview guide. During the analysis phase, inter-rater reliability was established through independent coding at the meaning unit level, where raw data were segmented and assigned to initial concepts. Two trained graduate researchers (one doctoral-level, one master’s-level) independently coded 20% of the data, focusing on: (1) identifying meaning units from raw transcripts, and (2) assigning these units to initial concepts. The Kappa coefficient of 0.85 was specifically calculated at the open coding level, measuring agreement on the assignment of meaning units to initial concepts, indicating strong inter-rater agreement. Discrepancies, which affected approximately 12% of the coded units, were systematically resolved through structured consensus meetings facilitated by the supervising faculty member, involving iterative discussion and reference to raw transcripts. This process ensured full consensus across all coded units. Ongoing calibration meetings were conducted throughout the coding process to maintain consistency. To further enhance transparency and reproducibility, a detailed coding rubric with explicit decision criteria is provided in Table S1 (Supplementary Materials), and representative coding examples across all categories are presented in Table S2.

#### 2.2.2. Validity

Multiple strategies were employed to enhance validity and trustworthiness, including member checking, peer debriefing via expert review, and methodological verification. Member checking was conducted with three strategically selected participants representing diverse educational backgrounds (Elementary school: N08; Middle school: N13; Associate degree or higher: D06). Participants reviewed preliminary coding results and thematic interpretations, and their feedback was systematically incorporated into the final analysis. Additionally, two independent experts in community psychology conducted a rigorous evaluation of the coding framework and analytical procedures, with their recommendations integrated into the final methodology. Data integrity was ensured through systematic documentation of transcription accuracy, and analytical process documentation to guarantee robust and defensible categorization procedures.

### 2.3. Results

#### 2.3.1. Composition of Common Prosperity Aspiration

The grounded theory analysis yielded 26 initial concepts derived from 328 meaning units, which were subsequently condensed into 12 sub-categories and ultimately grouped into four overarching categories: material–individual, material–collective, spiritual–individual, and spiritual–collective (see Table 2; see also Supplementary Table S1 for the full list of main categories and sub-categories with their connotations, and Supplementary Table S2 for illustrative coding examples). This theoretical model captures culturally distinct dimensions of common prosperity aspiration in rural Chinese communities, thereby addressing a gap in the operationalization of rural residents’ common prosperity aspirations.

Levels in responses were hierarchically distinguished using inductive rules and expert-guided criteria (see Section 2.1.3). Variations emerged across participant subgroups. Lower-income participants more frequently referenced basic material–individual needs (e.g., adequate food and clothing, housing improvement). Residents in the demonstration area showed relatively balanced references across all four categories, whereas residents in the non-demonstration area emphasized material dimensions. Village cadres placed greater emphasis on collective categories (e.g., infrastructure, public services) compared to ordinary residents, who prioritized individual-level improvements.

#### 2.3.2. Two-Dimensional Four-Quadrant Matrix Model

Using grounded theory, this study constructs the psychological connotation of rural residents’ common prosperity aspiration through continuous comparison of interview data from local village cadres and residents. The research reveals that the common prosperity aspiration is a multidimensional psychological construct, encompassing two orthogonal dimensions: material versus spiritual, and individual versus collective.

**Figure 1:**
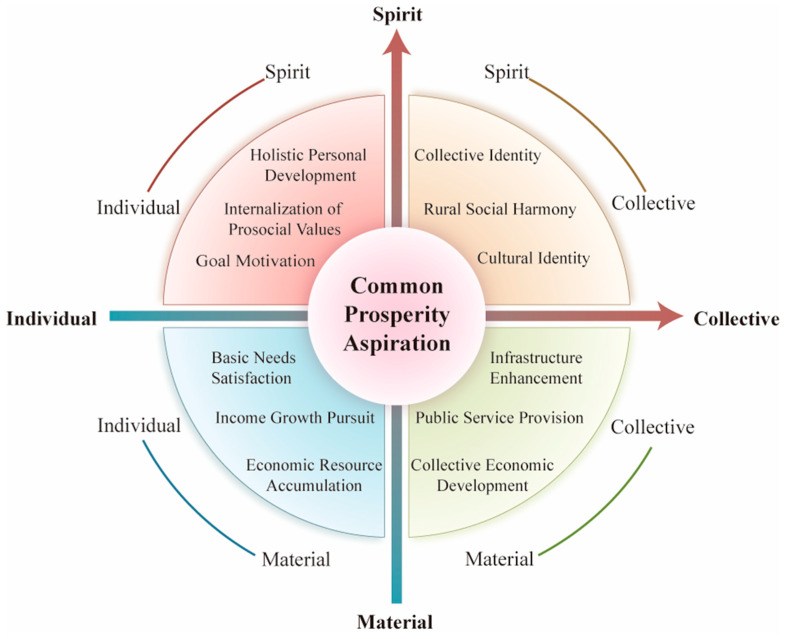
Two-dimensional four-quadrant matrix model of rural residents’ CPA.

These dimensions reflect individuals’ cognitive and motivational orientations toward comprehensive development (both material and spiritual) for themselves and their collectives. They interact dynamically and reinforce each other, collectively shaping individuals’ overall expectations for an enhanced quality of life. To systematically conceptualize this psychological construct, this study proposes a two-dimensional four-quadrant matrix model (see Figure 1), operationalizing the common prosperity aspiration into four distinct yet interconnected components: material–individual (MI), material–collective (MC), spiritual–individual (SI), and spiritual–collective (SC). Each quadrant represents a unique psychological orientation, as detailed below.

The MI quadrant reflects residents’ personal economic aspirations and self-directed efforts to improve their financial well-being, manifested in their pursuit of income-earning opportunities and autonomous economic behaviors. This component is operationalized through indicators such as satisfaction of basic needs, pursuit of income growth, and economic resource accumulation, which together constitute the primary motivation drivers for individuals seeking enhanced material living standards.

The MC quadrant emphasizes individuals’ prosocial orientation toward community economic development, particularly their commitment to supporting others’ prosperity and promoting shared growth. This component includes indicators of community infrastructure enhancement, public service provision, and collective economic development, demonstrating individuals’ collective efficacy beliefs and willingness to contribute to the communal material advancement.

The SI quadrant reflects individuals’ intrinsic motivation for self-actualization, highlighting their self-efficacy beliefs regarding personal development and positive future expectations. This component is assessed using indicators such as holistic personal development, internalization of prosocial values, and future-oriented achievement motivation, reflecting the psychological drivers for spiritual growth and the meaning-making process.

The SC quadrant focuses on individuals’ cultural identification and commitment to collective spiritual development. This component includes indicators of rural social harmony, collective identity, and cultural identity, reflecting individuals’ sense of belonging and active participation in the collective’s spiritual and cultural construction.

These four components are theoretically and empirically interdependent, forming an integrated psychological system. For instance, personal material accumulation can enhance collective well-being through positive spillover mechanisms, while supportive community environments and opportunity structures created by collective development reciprocally facilitate individual growth. At the same time, satisfaction of material needs provides the psychological foundation for higher-order spiritual pursuits, which, in turn, generate renewed motivation for material development. This bidirectional and cyclical relationship underscores the systemic complexity and holistic nature of the common prosperity aspiration construct; it aligns with the principle that lower-order needs provide the foundation for higher-order aspirations (Maslow, 1954) and that individual and collective orientations dynamically interact in collectivist cultural contexts (Markus & Kitayama, 1991).

## 3. Study 2: Initial Development of the CPAS for Rural Residents

### 3.1. Study Aim

Drawing from the qualitative findings of Study 1, which explored the psychological dimensions of common prosperity aspiration among rural community residents through in-depth interviews, this study aims to develop a preliminary measurement instrument based on the identified thematic patterns.

### 3.2. Method

#### 3.2.1. Initial Questionnaire Development and Item Validation

Based on qualitative interview data analyzed using grounded theory, an initial scale comprising 56 items was developed. To ensure content validity, the scale underwent expert evaluation and resident comprehension testing. In the expert evaluation phase, five experts (one psychology professor and four psychology graduate students) assessed each item for content coverage, clarity, relevance, and redundancy using a 5-point scale (1 = completely inappropriate, 5 = completely appropriate). Based on these evaluations, items with an average score below 3.5 were revised or eliminated. In the comprehension testing phase, 30 rural residents evaluated item clarity on a 5-point scale (1 = completely unclear, 5 = completely clear). Items with an average score below 4.0 or with more than 20% of respondents rating them below 3 were revised (Polit & Beck, 2017). Following two rounds of evaluation and research team discussions, seven items were eliminated (two for irrelevance, two for leading phrasing, and three for semantic redundancy), and two unclear items were revised. The resulting preliminary scale consisted of 49 items organized across the identified dimensions. The scale employs a 5-point Likert scale (1 = strongly disagree, 5 = strongly agree), with higher scores indicating higher levels of common prosperity aspiration.

#### 3.2.2. Participants

Data collection was conducted in July 2024 across rural communities in 18 provinces, autonomous regions, and municipalities in China. Participants were rural residents aged 18 and above. To account for the potential influence of “return migration” on rural development, the sample also included rural-origin university students with strong potential to return to their home villages after graduation and engage in rural development initiatives. A total of 770 questionnaires were distributed by trained surveyors. After cleaning (excluding questionnaires with more than 15% missing responses or completion times shorter than 2 s per item), 581 valid questionnaires were retained, yielding a response rate of 75.45%. The regional distribution of valid responses was as follows: eastern region (243 responses, 41.83%), central region (93 responses, 16.01%), and western region (245 responses, 42.17%). Among the valid respondents, 269 (46.30%) were male, with ages ranging from 18 to 79 years (*M*_age_ = 39.86, *SD* = 14.59), 397 (68.33%) were married, and 366 (62.99%) had high school education or below.

#### 3.2.3. Research Procedure

This study adhered to a standardized data collection process. To ensure data quality, all surveyors underwent standardized training to master the procedures. Prior to data collection, surveyors explained the study’s purpose, content, and expected completion time (15–20 min) to participants, emphasizing voluntary participation, anonymity, and confidentiality. Furthermore, the research team held regular discussions to address issues encountered during data collection. Upon completion, participants who completed the questionnaires received a small token of appreciation, such as hand sanitizer or soap.

#### 3.2.4. Data Analysis

The collected questionnaire data were analyzed using SPSS 27.0 and R 4.2.1. SPSS 27.0 was used for descriptive statistics, item analysis, reliability analysis, and exploratory factor analysis (EFA), while R 4.2.1 was employed for exploratory graph analysis (EGA). Although EFA is widely used in psychological research, its reliance on linear correlations limits its ability to capture potential nonlinear relationships (Fabrigar et al., 1999). Traditional objective criteria, such as relying solely on the eigenvalue-greater-than-one rule and scree plot examination, can lead to unstable dimensional identification (Goretzko et al., 2021). To address these limitations and obtain more robust evidence, this study used EGA, an emerging methodology, as a complement to traditional EFA approaches. EGA integrates network theory with data-driven community detection algorithms to identify complex linear and potentially nonlinear relationships among items. Recent empirical investigations have validated its superior reliability and effectiveness in dimensional assessment (F. Wang et al., 2025; Wu, 2024), with some researchers considering EGA as approaching the “gold standard” for assessing dimensions (Golino et al., 2020).

Data Quality Assessment. To assess normality of item distributions, skewness (|skewness| < 2) and kurtosis (|kurtosis| < 7) were examined for all items (Hair et al., 2010). Item quality was evaluated using two complementary methods: the critical ratio method and corrected item-total correlation (CITC). The critical ratio method assesses item discrimination by comparing extreme groups (top and bottom 27% of participants) via independent samples *t*-tests, while CITC evaluates item homogeneity and its contribution to overall scale reliability (Crocker & Algina, 1986; Ebel & Frisbie, 1991). Using both methods provides a more comprehensive assessment of item quality, as recommended in classical measurement texts (Crocker & Algina, 1986; Ebel & Frisbie, 1991). Items with CITC < 0.40 or whose deletion substantially increased Cronbach’s alpha were considered for elimination (Nunnally & Bernstein, 1994).

Exploratory Factor Analysis (EFA). EFA was conducted using SPSS 27.0 with principal axis factoring and Promax oblique rotation. Suitability for factor analysis was confirmed by the Kaiser-Meyer-Olkin (KMO > 0.7) measure of sampling adequacy and a significant Bartlett’s test of sphericity (*p* < 0.05) (Hair et al., 2010). Factor retention was primarily based on the convergence of parallel analysis (Horn, 1965; O’Connor, 2000), and the Minimum Average Partial (MAP) test (Velicer, 1976; O’Connor, 2000), supplemented by Kaiser’s criterion, scree plot inspection, and theoretical coherence (Hayton et al., 2004). Items were considered for elimination if their factor loading <0.40 or if the absolute cross-loading difference <0.20 (Hinkin, 1998). EFA was repeated iteratively until no items met the elimination criteria.

Exploratory Graph Analysis (EGA). Following Christensen and Golino (2021), EGA was conducted using the EGAnet package in R 4.2.1. First, network estimation was performed with the EGA function, which employs a Gaussian graphical model to capture conditional dependence among items and the Walktrap algorithm to identify community structures, thereby determining the number of dimensions. Second, the network was visualized using the Fruchterman-Reingold force-directed layout algorithm, positioning strongly connected items centrally to represent topological and connectivity patterns. Third, network stability was assessed using the bootnet package with a nonparametric bootstrap (1000 resamples) to compute 95% confidence intervals for edge weights. Stability was evaluated based on dimension detection frequency (>0.75) and item replication consistency (Christensen & Golino, 2021).

### 3.3. Results

Normality tests showed that the skewness values for all items ranged from −1.37 to −0.01, and kurtosis values ranged from −0.41 to 2.27, both within acceptable limits, suggesting the data satisfied the univariate normality assumption for parametric tests (Hair et al., 2010).

#### 3.3.1. Item Analysis

The critical ratio method showed that the absolute *t*-values for all items ranged from 9.17 to 17.67 (*p* < 0.001), confirming strong item discrimination (Ebel & Frisbie, 1991). For CITC, the corrected correlation coefficients between each item and the total score of its respective scale or dimension ranged from 0.35 to 0.71 (*p* < 0.001). Two items, t15 (*r* = 0.35) and t39 (*r* = 0.37), were eliminated due to failing to meet the criterion of *r* > 0.40 (Nunnally & Bernstein, 1994). After eliminating t15 and t39, the Cronbach’s alpha increased slightly from 0.954 to 0.955. As the change in Cronbach’s alpha was minimal and all remaining 47 items met the criteria for discrimination and homogeneity (*r* > 0.40, *p* < 0.001), no further items were eliminated. The final scale, comprising 47 items, demonstrated high internal consistency (α = 0.95).

#### 3.3.2. Exploratory Factor Analysis

EFA was conducted on a dataset (*N* = 581). Bartlett’s test of sphericity (χ^2^ = 14,158.10, *df* = 1081, *p* < 0.001) and the KMO test (KMO = 0.95) indicated that the data were suitable for factor analysis. Multiple criteria were used to determine the number of factors to retain. The Kaiser criterion (eigenvalue > 1) suggested 9 factors. Parallel analysis indicated that five factors had eigenvalues exceeding the corresponding 95th percentile values from random data. The original MAP test reached its minimum at the 5th factor, whereas the revised MAP test reached its minimum at the 3rd factor. The scree plot exhibited a sharp drop from the first to the second factor, followed by a more gradual decline, with the slope flattening noticeably after the second or third factor (see Table S3 for detailed results of parallel analysis, MAP tests, and scree plot).

Given the divergent results across methods, we evaluated solutions with 3 to 6 factors. The four-factor solution offered the best balance of statistical fit, theoretical interpretability, and parsimony, in line with recommendations for integrating multiple criteria (Hayton et al., 2004). Through iterative refinement, 28 items were removed across iterations primarily due to low primary loadings (<0.40) or substantial cross-loadings (difference from primary loading < 0.20). The retained items all exhibited primary loadings ≥0.50, ensuring a clean factor structure while preserving adequate representation of the core themes identified in Study 1 (cf. Comrey & Lee, 1992; Tabachnick & Fidell, 2019). This resulted in a final 19-item scale with a clear four-factor structure explaining 59.58% of the total variance. Factor loadings ranged from 0.56 to 0.92 (as shown in Table 3). The final CPAS for rural residents consisted of 19 items across four dimensions: MC (6 items), MI (5 items), SC (4 items), and SI (4 items). Cronbach’s α values were 0.84, 0.82, 0.82, and 0.80 for the respective dimensions, with an overall scale α of 0.90. Table 3 presents the factor loadings, communalities, and dimension assignments. All items showed adequate communalities (range: 0.47–0.80), and inter-item correlations within factors ranged from 0.34–0.77, below the 0.80 threshold for redundancy. Harman’s single-factor test revealed that the first unrotated factor explained 34.95% of variance, indicating minimal common method bias. The specific items and the Chinese version of the CPAS are provided in Supplementary Table S4.

#### 3.3.3. Exploratory Graph Analysis

**Figure 2:**
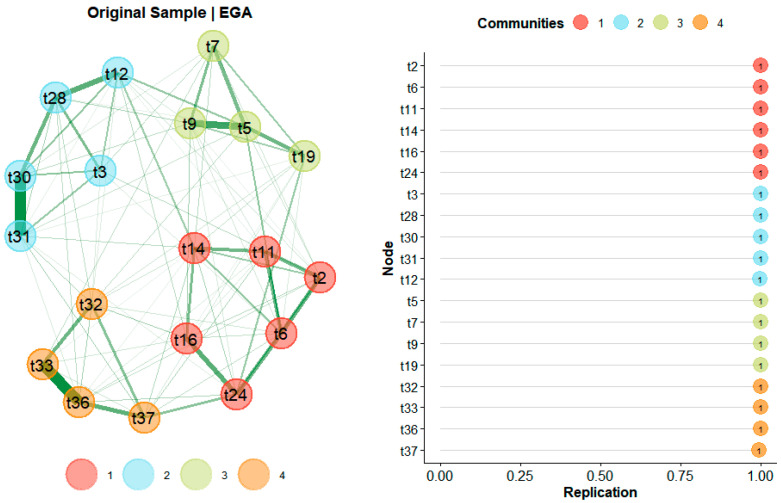
EGA results for the CPAS: (**left**) Network structure of the items, where nodes represent scale items, edge thickness indicates association strength (thicker for stronger), and edges are colored green for positive associations; communities are color-coded (1: red for MC, 2: light blue for MI, 3: green for SC, 4: orange for SI); (**right**) Bootstrap stability analysis, showing replication frequencies (all at 1.00) for each item across 1000 resamples, with colors matching communities.

EGA identified a four-dimensional network structure for the CPAS for rural residents, providing strong convergent evidence for the four-factor solution obtained from EFA and reinforcing our factor retention decision (see Figure 2, left). The dimensions were: MC (Items t11, t2, t6, t14, t24, t16), MI (Items t30, t31, t28, t3, t12), SC (Items t33, t36, t32, t37), and SI (Items t5, t9, t7, t19). Nodes represent the 19 scale items, with edge thickness indicating the strength of associations (thicker edges denote stronger associations) and edge colors representing the nature of associations. The bootEGA method (1000 resamples) detected the four-dimensional structure in 99.60% of bootstrap samples, with all items correctly classified into their theoretical dimensions (frequency = 1.00) and structural consistency coefficients of 1.00 for each dimension (see Figure 2, right). This high bootstrap detection rate (99.60%) underscores the exceptional stability and replicability of the four-dimensional structure, suggesting that the CPAS dimensions are robust against sampling variability and thus reliable for assessing rural residents’ aspirations in diverse community contexts. Furthermore, the perfect item classification and structural consistency (1.00) indicate minimal ambiguity in dimension assignments, reinforcing the theoretical distinction between material/spiritual and individual/collective orientations as proposed in this study. Network loadings, indicating standardized item strength, exceeded the recommended threshold of 0.15 for all items (see Table S5 in the Supplementary Materials), corresponding to at least moderate factor loading levels (≈0.40; Christensen & Golino, 2021). These loadings confirm strong item-dimension associations, ensuring that each subscale meaningfully captures its intended construct. Overall mean item response scores (*M* = 4.00, *SE* = 0.01, 95% CI [3.88, 4.12]) further indicated generally positive levels of common prosperity aspiration among rural residents. These results robustly support the four-dimensional network structure of rural residents’ common prosperity aspiration.

## 4. Study 3: Reliability and Validity Analysis of the CPAS

Building upon the CPAS for rural residents developed in Study 2, Study 3 seeks to validate the dimensional structure of the scale and assess its reliability and validity through a rural community sample.

### 4.1. Participants

To test the reliability and validity of the finalized scale, consistent with Study 2, data collection was conducted in August 2024, with all surveyors having received standardized training in data collection methods and ethics. The data collection procedures and data cleaning methods were consistent with those in Study 2 (see Section 3.2 for details).

Sample 1: For confirmatory factor analysis (CFA) and reliability and validity tests, a total of 885 questionnaires were distributed, of which 659 were valid, yielding an effective response rate of 74.58%. The sample’s geographical distribution covered eastern (159 responses, 24.13%), central (179 responses, 27.16%), and western China (321 responses, 48.71%). Among valid respondents, 324 (49.17%) were male, with ages ranging from 18 to 78 years (*M*_age_ = 41.35, *SD* = 15.70), 453 (68.74%) were married, and 413 (62.76%) had a high school education or below.

Sample 2: This subsample was drawn from Sample 1. For the test–retest reliability assessment, 74 retest questionnaires were distributed to the same rural residents two months later, yielding 62 valid responses (valid response rate = 83.78%). Among valid respondents, 29 (46.77%) were male, with ages ranging from 18 to 59 years (*M*_age_ = 33.16, *SD* = 14.23), 31 (50.00%) were married, and 21 (33.87%) had a high school education or below.

### 4.2. Instruments

#### 4.2.1. Common Prosperity Aspiration Scale

The Common Prosperity Aspiration Scale, developed in Study 2, is a formalized measure comprising 19 items across four dimensions (see Table S4 in Supplementary Materials). It employs a 5-point Likert scale (1 = strongly disagree, 5 = strongly agree), with higher total scores indicating greater common prosperity aspiration. The scale demonstrates high internal consistency reliability (Cronbach’s α = 0.91).

#### 4.2.2. Overall Well-Being Index Scale

The Overall Well-Being Index Scale (OWIS; Liu et al., 2012) measure an individual’s overall well-being. Comprising six items rated on a 5-point Likert scale (1 = strongly disagree, 5 = strongly agree), the OWIS demonstrates acceptable internal consistency reliability (Cronbach’s α = 0.77). Research shows it correlates strongly with material and spiritual dimensions of quality of life among community members (Bhattacharya, 2010).

#### 4.2.3. Striving Belief Scale

The Striving Belief Scale (SBS; Burgess, 2011), derived from the “effort leads to reward” dimension of the Social Axioms Scale, assesses belief in the causal link between effort and reward. The scale comprises four items, scored on a 5-point Likert scale (1 = strongly disbelieve, 5 = strongly believe), with higher scores indicating a stronger belief that effort leads to corresponding rewards. The SBS demonstrates acceptable to good internal consistency reliability (Cronbach’s α = 0.80). Prior studies link this belief to personality traits, motivation orientations, and universal driving forces (H. Chen et al., 2006).

#### 4.2.4. Community Identity Scale

The Community Identity Scale (CIS; Xin & Ling, 2015) measures individuals’ sense of belonging and recognition within their community. It comprises eight items across two dimensions, functional identity and emotional identity, using a 6-point Likert scale (1 = completely disagree, 6 = completely agree) and shows high internal consistency (Cronbach’s α = 0.90). The CIS correlates positively with public facility satisfaction, social support, and life satisfaction (Z. Zhang & Zhang, 2017). Reflecting material (e.g., community resources) and spiritual (e.g., emotional attachment) dimensions, it aligns with MC and SC dimensions of the CPAS.

#### 4.2.5. Prosocial Behavior Tendency Scale

The Prosocial Behavior Tendency Scale (PBTS; Carlo & Randall, 2002; adapted by Kou et al., 2007) is a 26-item scale that assesses prosocial behavior tendencies using a 5-point Likert scale (1 = very unlike me, 5 = very like me), with excellent internal consistency (Cronbach’s α = 0.94). Prosocial behavior tendencies are theoretically linked to aspirations for collective well-being, particularly in collectivist cultures where spiritual care and material support play key roles (An et al., 2024).

### 4.3. Research Procedures and Data Processing

The CPAS, OWIS, SBS, CIS, and PBTS were administered to Sample 1, and the CPAS was administered to Sample 2 at a two-month interval.

Structural validity of the CPAS was assessed through confirmatory factor analysis (CFA) using Mplus 8.3, with model fit indices and cut-off criteria identical to those reported earlier (L. Hu & Bentler, 1999; Kline, 2023). We proposed the following explicit a priori hypotheses regarding validity evidence based on relations to other variables:

**H1.**  *The CPAS total and subscale scores would show moderate to strong positive correlations with theoretically related constructs (SBS, CIS, and PBTS), providing convergent validity evidence. In particular, given the collectivist cultural embedding of the common prosperity aspiration construct in the Chinese context, we expected the two collective dimensions (MC and SC) to show significantly stronger correlations with prosocial behavior tendencies (PBTS) than the two individual dimensions (MI and SI).*

**H2.**  *The CPAS total and subscale scores would show moderate positive correlations with the OWIS, providing concurrent validity evidence.*

To determine the optimal factor structure, model fit was compared across one- to four-factor models, a bifactor model, and a second-order model. This examined the presence of a general common prosperity aspiration factor orthogonal to the four specific factors. Model optimization was guided by modification indices. Fit evaluation employed several indices: chi-square to degrees of freedom ratio (χ^2^/*df*), Comparative Fit Index (CFI), Tucker–Lewis Index (TLI), Standardized Root Mean Square Residual (SRMR), and Root Mean Square Error of Approximation (RMSEA). Acceptable model fit was determined using conventional criteria: χ^2^/*df* < 3, CFI > 0.90, TLI > 0.90, SRMR < 0.08, and RMSEA < 0.08 (L. Hu & Bentler, 1999). Given that χ^2^/*df* may increase with larger sample sizes and can be unstable, some studies accept χ^2^/*df* < 5 as adequate (Kline, 2023).

Measurement invariance across regions (eastern, central, western) was tested using multi-group confirmatory factor analysis with weighted least squares mean- and variance-adjusted (WLSMV) estimation for ordinal data. We examined configural (same factor structure), metric (equal factor loadings), and scalar (equal item thresholds) invariance sequentially. Invariance was evaluated using |ΔCFI| ≤ 0.01, |ΔTLI|≤ 0.01, and |ΔRMSEA| ≤ 0.015 as recommended by F. F. Chen (2007), as these indices are less sensitive to sample size than chi-square difference tests.

Reliability and validity analyses were conducted using SPSS 27.0. For reliability, we calculated McDonald’s omega coefficients (McDonald, 1999) for the total CPAS and its dimensions, along with internal consistency coefficients and test–retest reliability using Sample 2 after a two-month interval. These metrics were chosen to account for the scale’s multidimensional nature and heterogeneous loadings, providing a robust estimate of reliability (Dunn et al., 2014). Convergent validity was evidenced by correlations with the SBS, CIS, and PBTS. Concurrent validity was supported by correlations with the OWIS as a broad criterion measure.

### 4.4. Results

#### 4.4.1. Confirmatory Factor Analysis

CFA was performed on the CPAS using data from 659 participants in Sample 1. Model fit was compared across one- to four-factor models, a bifactor model, and a second-order model to examine the presence of a general common prosperity aspiration factor orthogonal to the four specific factors (see Table 4). The one-, two-, and three-factor models were tested as nested alternatives to assess parsimony and discriminant validity by merging theoretically related dimensions (e.g., material vs. spiritual, individual vs. collective; Brown, 2015). Poor fit was observed for the one-, two-, and three-factor models, whereas the four-factor model exhibited good fit (χ^2^/*df* = 4.64, CFI = 0.91, TLI = 0.89, SRMR = 0.05, RMSEA = 0.07). The second-order four-factor model showed comparable overall fit with identical incremental indices (χ^2^/*df* = 3.67, CFI = 0.91, TLI = 0.89, SRMR = 0.06, RMSEA = 0.06), but a significant chi-square difference test indicated superior parsimony (Δχ^2^ (2) = 150.102, *p* < 0.001). Standardized factor loadings for the selected second-order model are presented in Table 5 (Panel A: first-order loadings ranging from 0.60 to 0.87; Panel B: second-order loadings), with hierarchical omega for the general factor (ωH = 0.91) confirming its reliability (McDonald, 1999). These results support the multidimensional four-factor structure identified in Study 2 while justifying the use of a higher-order common prosperity aspiration construct. Although the bifactor model exhibited comparable fit, inspection of its standardized loadings revealed several near-zero or small negative values on the specific factors (ranging from −0.07 to 0.83), suggesting limited unique variance beyond the general factor.

#### 4.4.2. Reliability Analysis

Internal consistency was assessed using both Cronbach’s alpha and McDonald’s omega coefficients, with the latter preferred due to its less restrictive assumptions. For Sample 1, hierarchical omega for the general factor was 0.91, and omega coefficients for the four dimensions (MC, MI, SC, and SI) were 0.86, 0.87, 0.91, and 0.89, respectively. Corresponding Cronbach’s alpha values were 0.91 (total scale), 0.84 (MC), 0.84 (MI), 0.85 (SC), and 0.79 (SI), respectively, all exceeding 0.70, indicating good to excellent internal consistency (Kline, 2023). Test–retest reliability, evaluated in Sample 2 with 62 participants after a two-month interval, showed intraclass correlation coefficients for the total scale and the four dimensions (MC, MI, SC, and SI) of 0.78, 0.77, 0.76, 0.75, and 0.75, respectively (all *p* < 0.001), supporting good temporal stability.

#### 4.4.3. Validity Evidence Based on Relations to Other Variables

Following contemporary validity theory (AERA et al., 2014; Messick, 1995), we examined construct validity evidence for the CPAS based on its relations to other variables. Pearson’s correlation analyses were conducted to test hypotheses H1 and H2. First, convergent validity evidence (H1) showed moderate to strong positive correlations between the CPAS total and subscale scores and theoretically related constructs: SBS, CIS, and PBTS (*r* = 0.33–0.69, all *p* < 0.001), supporting H1 and aligning with theoretical expectations linking individual striving beliefs, social belonging, and altruistic tendencies to common prosperity aspirations. As anticipated within H1, the collective dimensions exhibited significantly stronger correlations with PBTS (MC: *r* = 0.63; SC: *r* = 0.61) than the individual dimensions (MI: *r* = 0.44; SI: *r* = 0.52; Steiger’s Z tests for dependent overlapping correlations, both *p* < 0.001), providing additional evidence for the collectivist cultural embedding of common prosperity aspirations in the Chinese context. Second, concurrent validity evidence (H2) revealed moderate positive correlations between the CPAS total and subscale scores and the OWIS (*r* = 0.30–0.44, *p* < 0.001), supporting H2. These findings, detailed in Table 6, collectively provide strong construct validity evidence for the CPAS through expected convergent and concurrent patterns, supporting hypotheses H1 and H2, and underscoring its scientific rigor and practical utility.

#### 4.4.4. Measurement Invariance Test

To examine whether the scale functions equivalently across the three regions, measurement invariance testing was conducted. Results are presented in Table 7. The configural invariance model demonstrated marginally acceptable fit (χ^2^ = 1248.79, *df* = 444, CFI = 0.87, TLI = 0.85, RMSEA = 0.091, SRMR = 0.072), confirming consistent factor structure across regions. Sequential constraints for metric invariance (equal factor loadings) and scalar invariance (equal item thresholds) were tested. The changes in fit indices remained within acceptable limits: metric invariance (χ^2^ = 1327.36, *df* = 480, ΔCFI = −0.007, ΔTLI = −0.004, ΔRMSEA = −0.001) and scalar invariance (χ^2^ = 1419.29, *df* = 508, ΔCFI = −0.010, ΔTLI = −0.002, ΔRMSEA = 0.001). These values met or approached established criteria (|ΔCFI| ≤ 0.010, |ΔRMSEA| ≤ 0.015; F. F. Chen, 2007), supporting scalar invariance. Given the ordinal nature of the indicators and WLSMV estimation method, strict invariance testing was not pursued, as this level is known to be overly restrictive for polytomous data (Svetina et al., 2020). The established scalar invariance provides sufficient evidence for valid cross-regional comparisons of latent means and structural relationships.

## 5. Discussion

### 5.1. Multi-Level Psychological Mechanisms of Common Prosperity Aspiration

Building on the qualitative findings from Study 1 and informed by cultural psychology, this study systematically investigates the psychological dimensions and structural components of rural residents’ CPA, defined as the psychological motivation to pursue personal fulfillment and collective advancement across material and spiritual domains within China’s rural revitalization context. Using a mixed-methods approach integrating qualitative and quantitative methods, we propose a two-dimensional four-quadrant matrix model that crosses material and spiritual goals (vertical axis), with individual and collective subjects (horizontal axis). This structure integrates Maslow’s (1954) hierarchy of needs to explain the material-to-spiritual progression and Markus and Kitayama’s (1991) interdependent self-construal theory to account for the prominent individual-to-collective variation, particularly highlighting the salience of collective dimensions in China’s collectivist rural settings.

Unlike prior research emphasizing economic or sociological perspectives (e.g., Dunford, 2022), this study focuses on rural residents’ psychological needs, particularly their integrated aspirations for personal and collective development. Traditional measures, such as Subjective Well-Being (Diener, 1984) and the Flourishing Scale (Diener et al., 2010), primarily assess individual emotional satisfaction and psychological outcomes, with limited capacity to capture the collective and material dimensions central to CPA. In contrast, CPA represents a motivational construct that drives the pursuit of personal economic goals, collective prosperity, self-actualization, and cultural engagement, embedded in rural China’s communal context. This reflects a dynamic interplay between individual and collective aspirations, aligning with Bronfenbrenner’s (1994) ecological systems theory, in which individual development is shaped by interactions across micro (family), meso (community), and macro (policy) levels.

The proposed model, validated through EFA, EGA, and CFA (Table 3, Table 5 and Table S5), elucidates multidimensional interactions: individual development supports collective well-being via spillover effects, while collective advancement provides resources for individual growth; similarly, material improvements enable spiritual elevation, and spiritual advancements foster material innovation. These processes operate across ecological levels (Figure 1). Correlation analyses (Table 6) further support the model: at the individual level, striving beliefs significantly correlate with MI and SI dimensions (*r* = 0.46; Hart, 2016); at the collective level, community identity strongly correlates with MC and SC dimensions (*r* = 0.46; McMillan & Chavis, 1986). Moreover, significant associations with striving beliefs, community identity, and prosocial behaviors confirm the CPAS’s criterion validity and its capacity to capture rural residents’ community-oriented aspirations. This multi-level mechanism model offers novel insights into the psychological foundations of rural community development under common prosperity goals.

### 5.2. Theoretical Contributions and Practical Implications

Grounded in community psychology and primarily informed by cultural psychology, this study advances the understanding of CPA in rural China. Theoretically, it conceptualizes rural residents’ CPA as a multidimensional psychological construct integrating material and spiritual dimensions at both individual and collective levels, building on prior research by integrating personal striving with collective efficacy and cultural identification in China’s common prosperity policy context. The four-dimension structure (MI, MC, SI, SC) extends Maslow’s (1954) hierarchy of needs, which accounts for the progression from material to spiritual aspirations, while incorporating Markus and Kitayama’s (1991) interdependent self-construal to explain the prominent collective orientation in rural Chinese collectivism. In collectivist settings, personal fulfillment is often embedded in relational harmony and shared progress, making collective aspirations (both material and spiritual, such as resource sharing and communal cultural identity) particularly salient. This culturally sensitive framework complements community psychology’s focus on collective efficacy and belonging, providing a more nuanced framework for how national policies are psychologically internalized in non-Western contexts.

Methodologically, the mixed-methods sequential design, combining qualitative grounded theory with quantitative analyses (EFA, EGA, and CFA), contributes to refinements in culturally grounded scale development. Measurement invariance testing across eastern, central, and western regions (|ΔCFI| < 0.01) supports the scale’s applicability across China’s diverse socioeconomic contexts. The established scalar measurement invariance further enhances the CPAS’s robustness, allowing valid cross-regional comparisons of latent scores despite socioeconomic differences. Although the item reduction during EFA (from 47 to 19 items) reflects a trade-off between parsimony and breadth, it resulted in substantially improved factor clarity and psychometric properties (e.g., high loadings ≥ 0.50, explained variance 59.58%) while fully retaining all core theoretical dimensions identified in Study 1. Convergent evidence from EGA (99.60% bootstrap stability) and CFA, along with the extensive initial item pruning common in indigenous scales developed in collectivist cultures, offers reassurance against sample-specific concerns and provides guidance for future instrument development in similar settings.

Practically, the validated CPAS provides actionable insights for community interventions and policy development within China’s rural revitalization framework. First, the CPAS equips policymakers with a tool to assess residents’ psychological aspirations across the four dimensions, MI, MC, SI, and SC, enabling targeted community development policies that enhance collective efficacy and cultural identification (Wandersman & Florin, 2003). For example, policies fostering community resource enhancement can strengthen residents’ sense of collective prosperity. Second, the CPAS serves as a reliable assessment tool for community intervention programs, allowing practitioners to track changes in residents’ psychological motivations and optimize interventions to support both material and spiritual needs. Third, the significant correlations between CPA and prosocial behaviors suggest that fostering such aspirations could promote community-oriented actions (e.g., participation in collective initiatives), thereby supporting broader common prosperity objectives.

### 5.3. Generalizability and Cultural Considerations

In this study, we developed and validated the CPAS within rural China, where collectivist norms, rooted in Confucian values of harmony (he), communal support (ren), and filial piety, profoundly shape aspirations for shared prosperity. These findings reflect rural China’s evolving political system, in which state-led policies such as hukou frameworks and rural revitalization programs reinforce collective aspirations, potentially explaining the stronger collective emphasis in our sample compared to more mobile urban contexts. This pattern echoes Fei’s (1992) description of rural societal fabric, and aligns with cultural psychological perspectives on self-construal (Markus & Kitayama, 1991), where interdependent self-views predominate in East Asian contexts and prioritize relational harmony and collective well-being over independent, individually focused aspirations. Such orientations make collective dimensions of prosperity (both material and spiritual) particularly salient in stable rural communities, whereas individual dimensions may dominate in contexts with greater migration flexibility, such as urban China or Western societies.

While the scale is tailored to these Chinese specificities, its four-dimensional structure (MI, MC, SI, SC) offers potential for cross-cultural dialogue. Supplementary interviews revealed urban-rural differences in manifestation. Rural residents emphasized communal efforts (e.g., “toilet revolutions” and “solar poverty alleviation”), whereas urban dwellers focused on individualized assets. Yet underlying psychological needs for security and belonging persist across settings. This aligns with theories on universal human needs (e.g., Deci & Ryan, 2000), adapted for collectivist contexts. This suggests that the framework could inform aspirations in other collectivist societies.

However, generalizability to non-Chinese contexts, such as Europe or Africa, requires caution. In individualistic European societies, the collective dimensions might be attenuated, prioritizing personal achievement over communal harmony (Hofstede, 1980). In African rural communities, where ubuntu philosophy underscores “I am because we are,” the scale might align more closely but would need adaptation for local economic and cultural factors (e.g., communal land tenure). Our sample’s homogeneity further limits broader applicability.

Future research should empirically test the CPAS’s applicability beyond rural China, perhaps through cross-cultural validations in regions like sub-Saharan Africa or Eastern Europe. Such studies could explore dimensional weightings (e.g., stronger individual-spiritual emphases in urbanized areas) and integrate community-based participatory methods for cultural fit. For reproducibility, researchers should retain the two-dimensional four-quadrant matrix as the core framework. The qualitative interview phase can be adapted to capture context-specific aspirations, whereas the quantitative validation stages provide a standardized approach to ensure psychometric rigor.

### 5.4. Limitations and Future Directions

Although this study offers valuable insights into the psychological needs underlying rural residents’ CPA, several limitations must be acknowledged. First, the sample’s representativeness is limited due to the lack of stratified sampling to account for regional variations (e.g., topographical or economic development differences) and the underrepresentation of younger respondents, influenced by rural aging trends (H. Wang & Chen, 2022). Future research should employ stratified sampling to examine CPA across diverse rural regions and age groups, thus enhancing generalizability within China. Second, measurement invariance across urban and rural populations remains untested. Rural communities often prioritize collective welfare, reflecting China’s differential mode of association values (Fei, 1992), whereas urban communities may emphasize individualistic orientations (Hofstede, 1980). Such differences could introduce measurement bias in cross-population comparisons. This cultural specificity may limit the scale’s applicability to other collectivist societies with different social structures or to Western contexts where community participation manifests differently. Future studies should conduct measurement invariance testing across rural–urban samples and pursue cross-contextual validations to ensure the CPAS’s applicability across rural, urban, and international settings. Third, the substantial item reduction during EFA (from 47 to 19 items, approximately 57%) may raise concerns about potential loss of content validity due to the elimination of a large portion of the initial item pool. This degree of reduction, while resulting in a parsimonious and psychometrically sound scale, reflects a common pattern in indigenous scale development in collectivist cultures, where shared cultural meanings often produce initial item overlap and semantic redundancy. Such redundancy can elevate inter-item correlations and increase overfitting risk if not addressed, and may also suggest potential method effects. The retention of all four core theoretical dimensions from Study 1, strong loadings (≥0.50), high explained variance (59.58%), exceptional EGA bootstrap stability (99.60%), and convergent CFA evidence help mitigate these concerns. Future research should include independent content validity assessments (e.g., content validity index), cognitive interviews, and cross-validation in new samples to further ensure comprehensive construct coverage and rule out sample-specific effects.

## 6. Conclusions

This study developed a theoretical framework and validated a psychometric instrument, the Common Prosperity Aspiration Scale (CPAS), for assessing Chinese rural residents’ psychological motivations to pursue common prosperity in rural community development. The findings indicate that common prosperity aspiration (CPA) is a multidimensional psychological construct, encompassing aspirations for material and spiritual fulfillment across individual and collective levels. This construct is represented by a two-dimensional four-quadrant matrix model based on the intersection of the material–spiritual dimension and the individual–collective dimension. The resulting 19-item CPAS measures four distinct dimensions (material–individual, material–collective, spiritual–individual, spiritual–collective) within a second-order factor structure, demonstrating robust psychometric properties, and providing a valuable tool for understanding rural residents’ psychological aspirations and supporting sustainable community development.

## Figures and Tables

**Table 1:** Demographic characteristics and background information of participants (*N* = 28).

ID	Gender	Age	Education	Marital Status	APCDI (¥)	SES	Region
N01 *	Male	32	High school	Married	12,000–20,000	6	NDA
N02 *	Male	41	Middle school	Married	2801–5000	5	NDA
N03 *	Male	43	High school	Married	5000–12,000	6	NDA
N04	Male	39	Middle school	Married	2801–5000	5	NDA
N05 *	Male	30	High school	Married	5000–12,000	5	NDA
N06	Male	45	Middle school	Married	<2800	5	NDA
N07 *	Female	28	Associate degree	Married	2801–5000	5	NDA
N08	Female	33	Elementary school	Married	2801–5000	6	NDA
N09	Female	57	Middle school	Married	<2800	5	NDA
N10 *	Female	50	Middle school	Married	2801–5000	6	NDA
N11 *	Female	51	Middle school	Married	<2800	5	NDA
N12 *	Female	25	Associate degree	Single	5000–12,000	5	NDA
N13	Male	28	Middle school	Single	5000–12,000	5	NDA
N14	Female	31	Middle school	Married	2801–5000	6	NDA
N15	Female	33	High school	Married	5000–12,000	5	NDA
D01 *	Female	52	High school	Married	20,000–30,000	6	DA
D02	Female	57	Associate degree	Married	20,000–30,000	6	DA
D03 *	Female	51	Associate degree	Married	>50,000	5	DA
D04	Male	54	Associate degree	Married	20,000–30,000	7	DA
D05 *	Male	59	High school	Married	30,000–55,000	6	DA
D06	Female	59	Associate degree	Married	12,000–20,000	4	DA
D07	Male	55	Associate degree	Married	12,000–20,000	6	DA
D08 *	Male	55	High school	Married	30,000–55,000	5	DA
D09	Male	63	Associate degree	Married	>55,000	6	DA
D10	Female	29	Associate degree	Single	20,000–30,000	6	DA
D11	Male	27	Associate degree	Married	20,000–30,000	6	DA
D12	Female	41	Bachelor’s degree	Married	>55,000	5	DA
D13	Male	42	High school	Married	>55,000	5	DA

*Notes*: * indicates village cadre; SES = subjective economic status (1–10 scale); NDA = non-demonstration area (western inland region); DA = demonstration area (eastern coastal region); APCDI = annual per capita disposable income. APCDI values are in Chinese Yuan (¥). For reference, China’s average annual per capita disposable income for rural residents in China in 2023 was approximately ¥21,691 (equivalent to about 3065 USD at an exchange rate of 1 USD ≈ 7.08¥); individual values can be approximately converted accordingly.

**Table 2:** Distribution of axial coding categories.

No.	Initial Open Code	References	Axial Coding Sub-Category	Main Category
1	Adequate food and clothing	14	Basic needs satisfaction	Material–Individual
2	Improvement in housing conditions	42		
3	Increased employment opportunities	21	Income growth pursuit	
4	Labor migration security	22		
5	Accumulation of production resources	10	Economic resource accumulation	
6	Disposable income	23		
7	Construction of water conservancy facilities	18	Infrastructure enhancement	Material–Collective
8	Agricultural facility security	31		
9	Imbalance in educational resources	17	Public service provision	
10	Shortage of medical resources	21		
11	Improvement in elderly care services	12		
12	Collective resource utilization	12	Collective economic development	
13	Industrial development and upgrading	11		
14	Physical health awareness	6	Holistic personal development	Spiritual–Individual
15	Intrinsic satisfaction from labor	5		
16	Moral self-discipline requirements	4	Internalization of prosocial values	
17	Self-behavior standards	5		
18	Enhancement of self-awareness	7		
19	Goal pursuit awareness	5	Goal motivation	
20	Personal development prospects	3		
21	Family harmony	5	Rural social harmony	Spiritual–Collective
22	Neighborly harmony	9		
23	Collective development concept	6	Collective identity	
24	Community identity	8		
25	Agricultural culture inheritance	4	Cultural identity	
26	Rural cultural construction	7		
	Total	328		

*Notes*: Initial open codes emerged from the open coding of meaning units in the raw transcripts. References indicate the number of unique meaning units (distinct meaning units coded to each node) across all interview transcripts. Axial coding sub-category groups these nodes into higher-level sub-categories derived from axial coding, illustrating the aggregation of initial concepts into thematic structures.

**Table 3:** Factor loadings and communalities from EFA of the 19-Item CPAS (*N* = 581).

Item	MC	MI	SC	SI	*h* ^2^
t11	0.86				0.57
t2	0.78				0.58
t6	0.78				0.65
t14	0.70				0.55
t24	0.64				0.56
t16	0.56				0.48
t30		0.89			0.73
t31		0.81			0.67
t28		0.75			0.60
t3		0.73			0.52
t12		0.62			0.50
t33			0.92		0.76
t36			0.90		0.80
t32			0.69		0.55
t37			0.61		0.53
t5				0.81	0.67
t9				0.77	0.63
t7				0.72	0.50
t19				0.72	0.47
Eigenvalues	6.64	1.90	1.58	1.21	
% of Variance	34.95	9.97	8.29	6.36	
Cumulative %	34.95	44.92	53.21	59.58	
Cronbach’s α	0.84	0.82	0.82	0.80	0.90 (Total)

*Notes*: MC = Material–collective; MI = Material–individual; SC = Spiritual–collective; SI = Spiritual–individual; Factor loadings < 0.50 are suppressed; *h*^2^ = communalities.

**Table 4:** Comparison of fit indices for different factor models of the CPAS (*N* = 659).

Model	χ^2^	*df*	χ^2^/df	CFI	TLI	SRMR	RMSEA
One-factor (MC + MI + SC + SI)	1414.07 ***	152	9.30	0.69	0.66	0.09	0.11
Two-factor (MC + MI, SC + SI)	1346.13 ***	151	8.91	0.71	0.67	0.09	0.11
Three-factor (MC, MI, SC + SI)	853.23 ***	149	5.73	0.82	0.80	0.09	0.09
Four-factor (MC, MI, SC, SI)	678.06 ***	146	4.64	0.91	0.89	0.05	0.07
Bifactor (G + MC + MI + SC + SI)	617.76 ***	133	4.64	0.91	0.89	0.06	0.07
Second-order (G on MC, MI, SC, SI)	527.96 ***	144	3.67	0.91	0.89	0.06	0.06

*Notes*: G = General factor (orthogonal to specific factors in the bifactor model; higher-order factor in the second-order model); “+” denotes factor merging; MC, MI, SC, and SI as previously defined; *** *p* < 0.001.

**Table 5:** Standardized factor loadings for the second-order model of the CPAS (*N* = 659).

(**A**) First-order factor loadings				
**Item**	**MC**	**MI**	**SC**	**SI**
t11	0.73			
t2	0.65			
t6	0.74			
t14	0.66			
t24	0.74			
t16	0.63			
t30		0.82		
t31		0.84		
t28		0.68		
t3		0.60		
t12		0.60		
t33			0.87	
t36			0.85	
t32			0.73	
t37			0.62	
t5				0.69
t9				0.72
t7				0.73
t19				0.66
(**B**) Second-order factor loadings				
**Path**	**Standardized Loading**			
General → MC	0.86			
General → MI	0.64			
General → SC	0.79			
General → SI	0.86			

*Notes*: All loadings are standardized and statistically significant (*p* < 0.001). MC, MI, SC, and SI as previously defined.

**Table 6:** Correlation between the CPAS and related validity variables (*N* = 659).

Variables	MC	MI	SC	SI	CPAS Total
CPAS Total	0.87 ***	0.71 ***	0.82 ***	0.81 ***	-
OWIS	0.32 ***	0.31 ***	0.30 ***	0.42 ***	0.44 ***
SBS	0.33 ***	0.37 ***	0.39 ***	0.43 ***	0.46 ***
CIS	0.37 ***	0.35 ***	0.39 ***	0.39 ***	0.46 ***
PBTS	0.63 ***	0.44 ***	0.61 ***	0.52 ***	0.69 ***

*Notes*: OWIS = Overall Well-Being Index Scale; SBS = Striving Belief Scale; CIS = Community Identity Scale; and PBTS = Prosocial Behavior Tendency Scale; MC, MI, SC, and SI as previously defined. *** *p* < 0.001.

**Table 7:** Tests of measurement invariance across three regions (*N* = 659).

Model	χ^2^	*df*	CFI	ΔCFI	TLI	ΔTLI	RMSEA	ΔRMSEA	SRMR
Configural	1248.79	444	0.87		0.85		0.091		0.072
Metric	1327.36	480	0.86	−0.007	0.85	−0.004	0.090	−0.001	0.086
Scalar	1419.29	508	0.85	−0.010	0.85	−0.002	0.090	0.001	0.089

*Notes*: Eastern region (*n* = 159), Central region (*n* = 179), Western region (*n* = 321).

## Data Availability

Qualitative data are not publicly available due to privacy restrictions imposed by the Institutional Review Board (IRB Approval No. H21092). Quantitative data supporting the findings of this study are openly available at https://www.scidb.cn/anonymous/bXlhSW5x (accessed on 26 May 2025).

## Supplementary Materials

The following supporting information can be downloaded at: https://www.mdpi.com/article/10.3390/bs16020203/s1, Table S1: Main categories and subcategories from selective coding of common prosperity aspirations. Table S2: Coding example of common prosperity aspirations. Table S3: Factor retention criteria for EFA. Table S4: Characteristic dimensions of the CPAS. Table S5: Item network loading estimated via EGA (*N* = 581).

## Abbreviations

The following abbreviations are used in this manuscript:

CPA	Common Prosperity Aspiration
CPAS	Common Prosperity Aspiration Scale
EFA	Exploratory Factor Analysis
EGA	Exploratory Graph Analysis
CITC	Corrected Item-total Correlation
KMO	Kaiser-Meyer-Olkin
MAP	Minimum Average Partial
CFI	Comparative Fit Index
TLI	Tucker–Lewis Index
SRMR	Standardized Root Mean Square Residual
RMSEA	Root Mean Square Error of Approximation
WLSMV	Weighted Least Squares Mean- and Variance-Adjusted
