# Supplementary material for: Development and Validation of the Common Prosperity Aspiration Scale: A Mixed-Methods Study in China
Source: Behav Sci (Basel). 2026 Jan 30;16(2):203. doi: 10.3390/bs16020203 (PMC12938126; doi:10.3390/bs16020203)
Supplement: Supplementary file 1 [file behavsci-16-00203-s001.zip › behavsci-3928241-supplementary.pdf]

behavioral sciences

MDPI

Supplementary Materials

Table S1. Main categories and subcategories from selective coding of common prosperity aspirations.

Main category	Sub-category	Connotation
Material – Individual	Basic needs satisfaction	It includes not only having enough food and clothing, but also the improvement of housing conditions, reflecting the transformation of rural life from survival to development.
	Income growth pursuit	It includes not only the increase of local employment opportunities, but also off-farm work or migrant labor, such as working outside the village, reflecting villagers' pursuit of economic autonomy and life improvement.
	Economic resource accumulation	It includes not only the accumulation of agricultural production materials, but also the pursuit of wealth accumulation with increased disposable income, reflecting the transformation of rural residents from merely making a living to achieving stable economic development.
Material - Collective	Infrastructure enhancement	It includes not only the construction of water conservancy facilities (e.g., irrigation channels, reservoir construction), but also the improvement of agricultural and transportation infrastructure (e.g., rural road and bridge repair), providing basic support for rural production, life, and economic development.
	Public service provision	It includes not only policy support and resource allocation, but also rural residents' fair access to basic public services such as education, medical care, and elderly care, reflecting the transformation from resource scarcity to the equalization of public services.
	Collective economic development	It includes not only the utilization of collective resources (e.g., land transfer), but also industrial upgrading (e.g., under-forest economy), reflecting the transformation to modern collective economy.
Spiritual - Individual	Holistic personal development	It includes not only the concept of physical health (e.g., regular physical examinations), but also the internal satisfaction of labor (e.g., the sense of achievement in farming), reflecting the transformation from a single survival need to a comprehensive healthy life.
	Internalization of prosocial values	It includes not only the requirement of moral self - discipline (e.g., being honest and kind), but also behavioral self - regulation (e.g., personal sense of responsibility), and the improvement of self-

 https://doi.org/10.3390/bs16020203

		awareness (e.g., self-reflection), reflecting the transformation of rural residents from external norm constraints to internal value consciousness.
	Goal motivation	It includes not only the awareness of goal pursuit (such as setting and completing goals), but also personal development prospects (such as mastering new skills to adapt to modern agriculture), reflecting the transformation from short-term livelihood maintenance to long-term self-realization.
	Rural social harmony	It includes not only family harmony (such as smooth parent-child communication), but also neighborhood harmony (such as neighborhood mutual assistance), reflecting the pursuit of harmonious social relations.
Spiritual- Collective	Collective identity	It includes not only the consensus on collective development (such as supporting collective projects), but also the sense of community identity (such as participating in village affairs activities), reflecting the transformation from scattered individuals to a cohesive community.
	Cultural identity	It includes not only the inheritance of farming culture (such as traditional planting), but also cultural innovation (such as rural tourism), reflecting the integration and recognition of both traditional and modern cultural elements.

Table S2. Coding example of common prosperity aspirations.

Original data	Open coding	Sub - category	Main category
N04: Regarding food, it's okay. Not to mention having very good food, at least there are things at home that can be prepared. Eventually, there's no problem with food and clothing.	Adequate food and clothing	Basic needs satisfaction	Material- Individual
N06: Of course, this is hoping that we live a better life. For example, every household hopes that every family has a house built. But now, some families here still live in tile-roofed houses.	Improvement in housing conditions		
D13: For example, it means that we have (work) opportunities here, and everything here has money. Hope it's like this.	Increased employment opportunities	Income growth pursuit	

https://doi.org/10.3390/bs16020203

N01: For labor output, if one can go out to work, the income will be different, and one can earn money, and can continuously earn money from working.	Labor migration security		
N05: You say that in such dry weather, the land can't be tilled (can't grow crops). If we get a few more cows to raise and sell, it's not bad, there's still something.	Accumulation of production resources	Economic resource accumulation	
D06: Also, in terms of life, there is a bit of savings.	Disposable income		
N08: The expectation is that in this village, in the future, the water problem can be solved.	Construction of water conservancy facilities	Infrastructure enhancement	
N05: Build farm-to-market roads. The farm-to-market road is now impassable and relatively far. If you open up all the roads, cars can drive to the side of the fields.	Agricultural facility security		
D03: There is a polarization. The good get better, and the bad get worse. Now it's not that the teaching resources are bad, but that the student resources are bad. The more powerful ones will send (their children) to better places.	Imbalance of educational resources		
N06: You mentioned medical care. If the condition is even slightly serious, we have to transfer the patient to another hospital. So, the medical care here isn't very good either—even minor issues get referred to the city hospital.	Shortage of medical resources	Public service provision	Material- Collective
N01: National policies are good, the elderly receive pensions in their old age. However, the aging problem remains serious. In the future, if conditions allow, the village will set up a canteen for them."	Improvement in elderly care services		
D05: In our rural areas, how to utilize resources and improve to a high efficiency involves two aspects, one is agriculture and the other is forestry (D05).	Collective resource utilization	Collective economic development	

https://doi.org/10.3390/bs16020203

D07: For example, we introduce a large-scale processing enterprise, and then upgrade the processing of products.	Industrial development and upgrading		
N04: The awareness of physical health is too weak. It's not because of lack of money, but they just won't do a check-up.	Physical health awareness	Holistic personal development	Spiritual- Individual
D02: Develop a mindset that since we go to work today, it's a kind of pleasure.	Intrinsic satisfaction from labor		
N10: You shouldn't think about crooked ways. For example, a gentleman should obtain wealth in a proper way and not do illegal things through crooked ways!	Moral self-discipline requirements	Internalization of prosocial values	
D01: That is, I can be worthy of myself and my conscience.	Self-behavioral standards		
D08: To see far is an improvement in terms of thinking or vision.	Enhancement of self-awareness		
N07: Isn't it because you don't work hard? You are just lazy and don't say what you want to accomplish. In a word, that's it.	Goal pursuit awareness	Goal motivation	
D07: Work hard steadily. Personally, it will definitely get better and better.	Personal development prospects		
N10: Husband and wife are harmonious, children are filial, life is comfortable, and everything is easy to discuss.	Family harmony	Rural social harmony	Spiritual- Collective
N04: The elderly and children get along harmoniously, and neighbors must get along harmoniously. When there is something, everyone can help each other.	Neighborhood harmony		
D05: Build our beautiful village, right? Build up the environment, economy, and even agriculture, forestry, and agricultural water and other aspects.	Collective development concept	Collective identity	

https://doi.org/10.3390/bs16020203

D06: We definitely don't have the (good conditions) like cities, but in terms of air quality, intelligent lighting, and landscapes. How is it worse than the city?	Community identity	
D09: Now, whether it's agriculture or under - forest economy, or ensuring the bottom line of food production, there must be people who love the field and love agriculture to manage it.	Agricultural culture inheritance	Cultural identity
D02: In our village, we hold a She ethnic festival every March 3rd to celebrate this festival. There is a cultural festival, and everyone can go on stage to perform and have a great time.	Rural culture construction	

Note: The numbers in brackets are the codes of the interviewees.

Table S3. Factor retention criteria for EFA.

Factor	Eigenvalue	Cumulative %	PA 95th percentile	SEscree	MAP power 2	MAP power 4
0					0.106	0.016
1	15.528	33.037	1.668	**2.033**	0.014	0.001
2	2.908	39.226	1.585	0.329	0.012	0.001
3	2.050	43.587	1.533	0.237	0.011	**0.000**
4	2.015	47.873	1.490	0.205	0.010	0.000
5	1.811	51.727	**1.452**	0.155	**0.008**	0.000
6	1.396	54.698	1.417	0.098	0.008	0.000
7	1.196	57.241	1.385	0.070	0.008	0.000
8	1.020	59.411	1.357	0.052	0.009	0.000
9	**1.004**	61.547	1.326	0.045	0.009	0.000
10	0.888	63.437	1.326	0.045	0.009	0.000

Note: Bold indicates meeting the respective criteria (eigenvalue > 1; eigenvalue > PA 95th percentile; first occurrence of MAP minimum, and SE > threshold for the SEscree test).

Table S4. Characteristic dimensions of the CPAS.

**Dimension (维度)**	**Item (条目)**	**Strongly disagree** 完全不同意	**Somewhat disagree** 比较不同意	**Neutral** 中立	**Some-what agree** 比较同意	**Strongly agree** 完全同意
Material-Collective 物质一集体	t11 Helping others achieve prosperity fosters mutual economic benefits.<br>t11 带动别人富裕是双赢的事情。<br>t2 I am willing to support others in achieving prosperity within my capacity.<br>t2 力所能及的情况下我愿意带动他人富裕。<br>t6 I am willing to provide resources and opportunities to help others enhance their income.<br>t6 我乐意提供资源和机会，帮助他人提高收入。<br>t14 Helping others achieve material wealth contributes to my own economic prosperity<br>t14 帮助别人获得物质财富的过程中，我也会变得更富裕。<br>t24 I am willing to invest time and effort to support others' economic prosperity<br>t24 我愿意花费时间和精力帮助他人致富。<br>t16 I advocate for shared prosperity among others.<br>t16 我向他人宣传共同富裕的理念。					
Material-Individual 物质一个人	t30 Even when fatigued, I persist in working hard to earn income.<br>t30 即使再累，我也会继续努力挣钱。<br>t31 Despite challenges in achieving financial gain, I remain persistent.<br>t31 即使赚钱路上困难重重，我仍坚持不懈。<br>t28 I consciously seek out opportunities to make money.<br>t28 我有意识地去关注挣钱的机会。<br>t3 I am willing to invest time and effort in pursuing economic opportunities.<br>t3 我愿意把更多的时间和精力投入到挣钱的事情上。					

https://doi.org/10.3390/bs16020203

	t12 I am capable of exploring diverse economic opportunities.<br>t12 我能尝试各种挣钱的机会。
Spiritual-Collective 精神一集体	t33 I participate in volunteer service activities.<br>t33 我会参加志愿者服务活动。<br>t36 I am enthusiastic about engaging in public welfare and volunteer activities.<br>t36 我热衷于参与社会公益和志愿服务。<br>t32 Organizing or participating in collective cultural activities makes me happy.<br>t32 组织或参与集体文化活动使我感到开心。<br>t37 I value contributing to others' well-being.<br>t37 我喜欢做有利于他人幸福的事。
Spiritual-Individual 精神一个人	t5 I believe I can realize my personal value.<br>t5 我相信我能够实现自己的价值。<br>t9 I believe I have the ability to create a hopeful future.<br>t9 我相信自己有能力创造充满希望的未来。<br>t7 My life has purpose and meaning.<br>t7 我的生活有目标、有意义。<br>t19 I am capable of fostering a sense of security in life and work.<br>t19 我觉得自己有能力在生活和工作中创造安全感。

Table S5. Item network loadings estimated via EGA (N = 581).

Item	Material-Collective	Material-Individual	Spiritual-Collective	Spiritual-Individual
t6	0.36			
t2	0.30			
t14	0.29			
t24	0.28			
t11	0.26			
t16	0.23			
t30		0.49		
t31		0.36		
t28		0.34		
t3		0.24		
t12		0.23		
t5			0.42	
t9			0.34	
t7			0.25	
t19			0.22	
t36				0.50
t33				0.44
t37				0.22
t32				0.22

https://doi.org/10.3390/bs16020203
